# Political Differences in Past, Present, and Future Life Satisfaction: Republicans Are More Sensitive than Democrats to Political Climate

**DOI:** 10.1371/journal.pone.0098854

**Published:** 2014-06-05

**Authors:** David R. Mandel, Philip Omorogbe

**Affiliations:** 1 Socio-Cognitive Systems Section, Defence Research and Development Canada, Toronto, Ontario, Canada; 2 Department of Psychology, York University, Toronto, Ontario, Canada; 3 Department of Psychology, Neuroscience and Behaviour, McMaster University, Hamilton, Ontario, Canada; Saarland University, Germany

## Abstract

Previous research finds that Republicans report being happier or more satisfied with their lives than Democrats. Using representative American samples from 2002, 2005, 2007, 2009, and 2010, we tested a Person × Situation interactionist account in which political affiliation (Democrat, Republican) and political climate (favorable when the president in office is of the same party) are proposed to affect past, present, and anticipated future life satisfaction. Meta-analyses of related tests of key hypotheses confirmed that (a) life satisfaction was greater when the political climate was favorable rather than unfavorable and (b) Republicans were more sensitive to political climate than Democrats. As predicted, Republicans also were more politically polarized than Democrats. Taken together, the findings indicate that, compared to Democrats, Republicans are more apt to self-identify in political terms, and core aspects of their subjective well-being are more easily affected by the outcome of political events.

## Introduction

A good life involves not only the lessening of suffering, but also the promotion and experience of happiness or life satisfaction, among other elements of subjective well-being [1], [2]. Studies using representative samples of Americans have shown that, compared to respondents who identify as liberal or Democrat, those who identify as conservative or Republican report being happier or more satisfied with their lives [3]–[6]. This conservative/Republican advantage in life satisfaction is evident even when demographic variables, such as sex, age, education, marital status, and income are statistically controlled [4], [5].

Alternative explanations of this conservative happiness boost have been proposed. For instance, drawing on system justification theory [7], it has been shown that the happiness boost for conservatives is mediated by system-justifying beliefs [4]. Furthermore, it has been proposed that such beliefs protect conservatives, in particular, from the harsh realities of living in an unequal world and supporting policies that reinforce inequality [4]. By contrast, others have argued that the effect is merely a facet of a more robust pattern of heightened subjective well-being in conservatives compared to liberals. For instance, compared to liberals, conservatives express greater personal agency, a more positive outlook, and stronger transcendent beliefs [5]. And, compared to Democrats, Republicans report fewer negative life events and crying episodes, and they report having more good friends and reliable social-support members [8]. Moreover, people with more good friends and better social support report being happier [9], [10]. In fact, socio-economic status (SES) directly predicts the number of individuals' group memberships [9]. Controlling for SES and number and intensity of group memberships, political orientation had virtually no remaining causal effect on life satisfaction. Contrary to [4], [9] also found that system-justifying beliefs predicted less life satisfaction.

### The Person × Situation Interactionist Account

Explanations of the conservative happiness boost differ in terms of their proposed mechanisms and functional implications. However, all share in common that they are person-level accounts. In this article, we shift the emphasis of this research topic somewhat by examining the effect of political group differences in life satisfaction in terms of a Person × Situation (P×S) interactionist account. Our account is not inconsistent with person- or group-level explanations, and may in fact be used to refine such accounts by elucidating important moderators of group-level differences. Indeed, several scholars have argued that theories of subjective well-being ought to pay greater attention to the interactions among individual-difference and situational factors [1], [11], reflecting a broader theoretical move towards P×S interactionism in personality and social psychology [12]–[14].

In the present context, we propose that interactionism can enrich our theoretical understanding of how individuals' life satisfaction as a core component of their subjective well-being is affected by their political identities. Our theoretical approach is congruent with cognitive-affective systems theory [13]. In that account, individuals encode features of situations in representationally meaningful terms, and such features may also activate and influence cognitive and affective reactions in those individuals. The reactions individuals experience in response to representationally encoded situations, in turn, may be moderated by individual differences, which may also be correlated with particular group memberships. In this paper, we propose that *political climate* is a situational factor defined by objective political facts, such as which political party is actually in power, which are meaningfully encoded by individuals. For instance, if the state leader is one that the individual supports, then the political climate will in all likelihood be viewed more favorably than if that leader was one the individual opposes. Moreover, political climate, as a representationally encoded situational factor, may activate and influence individuals' cognitive and affective reactions, such as their feelings or evaluations of life satisfaction. Those affective and cognitive responses may, in turn, be predictably moderated by individual-level (e.g., political orientation) or group-level (e.g., party affiliation) differences.

The first premise of our account is that partisans encode presidential electoral outcomes as favorable or unfavorable depending on whether the political victor shares their party affiliation (favorable) or does not (unfavorable). In the US context, which we focus on here, we define political climate as favorable if the individual and president in power (or recently elected) are both Democrats or are both Republicans. Political climate is unfavorable if one is a Democrat and the other is a Republican. For nonpartisan Independents, we define political climate as invariably intermediate. Although we acknowledge that political climate may be shaped by Congressional outcomes, we suspect that, for most citizens, the results of those outcomes represent more of a bounding or freeing of presidents to implement their vision. Partisan voting is also considerably weaker in Congressional elections than in presidential elections [15]. Moreover, Americans are more likely to correctly identify presidents than Congressional leaders, such as the Speaker of the House [16]. Accordingly, we focused on partisan congruence or incongruence with presidential power as a basis for defining political climate.

As noted earlier, political climate is not a purely situational factor, but rather a representationally encoded aspect of political losses and victories, which is defined in relation to political group differences (namely, political affiliation). Political climate is therefore a P×S factor. Our first hypothesis is that US partisans will report greater life satisfaction in the present if they are in a favorable political climate than if they are in an unfavorable climate. A corollary of this *favorability hypothesis* is that Independents, on average, will report a level of life satisfaction between those experiencing favorable and unfavorable climates. Thus, we predicted an ordered main effect of political climate on present life satisfaction.

Prior studies addressing this question have yielded mixed and ambiguous findings. [4] reported that happiness was not significantly predicted by the interaction of political conservatism and the party in power in General Social Survey (GSS) data. However, GSS respondents were asked about their happiness after having been extensively queried about their political views and behavior. It is well established that the ordering of survey questions can have large effects on responses [17]. It is unclear what the effect of assessing one's happiness in general *after* having answered a large number of political, social, and economic questions may be. Clearly, it would be preferable to ask respondents about their life satisfaction first, before they are cued or primed by other questions and their responses to them. Although it is conceivably possible that respondents' responses to the political affiliation question were affected by their life satisfaction assessments, it strikes us as much more probable that getting people to think about their political affiliations would influence their assessments of life satisfaction.

As well, there is much convergent support for the favorability hypothesis. For instance, partisans think wishfully about electoral outcomes [18] and they are more likely to vote if they find one candidate favorable and another unfavorable [19], suggesting that such outcomes carry hedonic weight in their lives. Partisans' satisfaction with the economy is also significantly influenced by whether the president in power shares their party affiliation [20], [21] (see [22] for comparable UK findings). In a related vein, Democrats reported becoming happier, while Republicans reported becoming less happy, immediately after the 2006 Democratic takeover of Congress [23]. However, in that study, respondents were surveyed in a narrow window before and after the election. It is unclear to what extent that pre-post design explicitly cued predictable partisan responses or how long the partisan happiness boost might have lasted. The present research used Pew Research Center survey data, which had the advantage of asking respondents about their life satisfaction at the outset of the survey, with different respondents being surveyed across years. Moreover, each year that we examined was at least one year after the last presidential election.

A second, key hypothesis of ours was that political affiliation and political climate would affect life satisfaction in an interactive manner. Specifically, we predicted that the effect of political climate on life satisfaction would be greater among Republicans than among Democrats. This represents a (P×S) ×P prediction that we call the *asymmetric sensitivity hypothesis*. Support for this hypothesis is based on multiple lines of evidence. First, studies show that conservatives are more sensitive than liberals to affective information in their environment. Compared to liberals, conservatives are more sensitive to disgust-inducing stimuli [24], [25], they pay more attention to affective information in their perceptual environment and have more difficulty inhibiting the effect of such information on their responses [26], they show stronger neurophysiological responses to threat stimuli [27], and they orient faster and spend longer attending to aversive stimuli [16]. Conservatives also anticipate that they will experience greater negative affect in response to negative outcomes and they do in fact experience greater negative affect [28]. Thus, Republicans might savor political wins and dread political losses more than Democrats.

The asymmetric sensitivity hypothesis is also supported by research indicating that Republicans have stronger political identities than Democrats. Part of this is owing to liberal-conservative differences. Compared to liberals, conservatives are more accurate in their stereotypes of both conservatives and liberals [29]. This difference in stereotype bias for both the political ingroup and outgroup suggests that, compared to liberals, conservatives pay greater attention to information about politics and process it more deeply. Conservatives also have stronger group-centered moral values than liberals [29], suggesting that they may be more sensitive to the favorability of political outcomes, given that such outcomes decide whether their political party will govern their country for the next four years. As well, whereas liberals have approach-oriented values, conservatives are avoidance-oriented, placing greater emphasis than liberals on the goal of reducing their own group's threat [16], [30]. Republicans, therefore, may be more inclined than Democrats to view a lost election as a significant threat to their future.

Several studies also indicate that, compared to liberals, conservatives have a lower tolerance of ambiguity and value conflict and, conversely, a stronger preference for certainty [31]–[33], including in the political domain [34]. We consider such evidence in light of the fact that, although conservatism is usually measured on a unidimensional, bipolar scale, evidence suggests that liberalism and conservatism are dissociable, yet moderately (negatively) correlated dimensions [35], [36]. Individuals who blend elements of liberalism and conservatism would thus score closer to the midpoint of a traditional conservatism scale than those who accept one political orientation but reject the other. Consistency-seeking Republicans, we predicted, would therefore place themselves further right of center than pluralism-tolerant Democrats would place themselves to the left. In other words, according to this *asymmetric political extremity hypothesis*, Republicans are more politically polarized than Democrats, being less inclined to blend liberalism into their political identity than Democrats would be to blend conservatism into theirs.

### Life Satisfaction: Past, Present, and Future

In the present research, we drew on a large, representative sample of Americans polled in the Pew Research Center's Global Attitudes Project and collected from 2002–2010. We focused on these datasets because they provided measures of life satisfaction for the past (5 years before the survey date), present (at the time of the survey), and future (5 years after the survey date) on a common scale, thus permitting multiple tests of the favorability and asymmetric sensitivity hypotheses, as well as the opportunity to meta-analyze the findings of those tests. For example, it has been shown that political climate affects optimism regarding financial markets [37]. Democrats were more optimistic than Republicans in 1996–1998 under the Clinton administration, but Republicans were more optimistic than Democrats in 2001–2002 under the Bush administration. No study, however, has examined the effect of political climate on more general assessments of future life satisfaction. Because anticipated future life satisfaction could conceivably be influenced by many factors other than politics, and because satisfaction measures in the Pew data sets were taken well in advance of any political measures being taken, the present study provides a conservative test of the influence of political climate on optimistic assessments.

Assessments of past life satisfaction provided a unique opportunity to test the effects of present and past political climates on life satisfaction. That is, not only could we examine past life satisfaction as a function of the respondent's present political climate, we could also examine it as a function of the political climate that existed 5 years prior. In formulating our predictions, we drew on the inclusion/exclusion model of evaluative judgment [38]. According to the model, information used to form a representation of an evaluative target results in assimilation effects, whereas information used in forming a representation of an evaluative standard results in contrast effects. In the present context, the evaluative target is past life satisfaction, and the information that would be used to form that representation includes the political climate that existed at that time. The political climate at the time of the survey, however, would be included in information that serves as the basis for an evaluative standard (i.e., “how satisfied am I with my life at present?”). Accordingly, we predicted that past life satisfaction would be an assimilative function of the past political climate and a contrastive function of the present. In both cases, however, we expected the asymmetric sensitivity hypothesis to moderate these effects. That is, we expected Republicans to show stronger assimilation and contrast effects than Democrats.

### Hypotheses

To summarize, we tested the following hypotheses: First, we tested the favorability hypothesis, which predicts an ordered effect of political climate such that those in a favorable climate will be more satisfied than those in an intermediate climate (i.e., Independents), who will be more satisfied than those in an unfavorable climate. Second, we tested the asymmetric sensitivity hypothesis, which predicts that the putative favorability effect will be stronger for Republicans than for Democrats. That prediction should be manifested as a significant political affiliation by political climate interaction effect, where the simple effect of political climate is stronger for Republicans than it is for Democrats. Those effects, moreover, are predicted to be consistent with assimilative processing in all cases except for past life satisfaction coupled with present political climate, where instead a contrastive effect based on the inclusion/exclusion model [38] is expected. Finally, we tested the asymmetric political extremity hypothesis, which predicts that Republicans locate themselves much further toward the conservative extreme on a political orientation scale than Democrats locate themselves toward the liberal extreme.

As noted earlier, we favor the use of the Pew data sets because they provide a conservative test of the effect of political affiliation, political climate and the interaction of those factors on life satisfaction measures, which were taken at the start of the surveys. Because we fully expect life satisfaction to be influenced by many factors other than the political ones unobtrusively studied in this research, we accordingly expected to observe small, perhaps even very small, effect sizes in inferential tests involving life satisfaction measures when assessed in terms of frequently used evaluative conventions [39]. However, we share the view of [40] and indeed [39] that just as statistical significance is a fallible guide to theoretical or practical importance, so are effect size conventions, which can provide only the roughest of guides regarding importance. In this research, given the subtlety of the elicitation of life satisfaction in relation to political affiliation, and the non-elicited basis of our key variable (political climate), we encourage the reader to adopt a contextualized appreciation of the reported findings.

## Method

### Participant Data

We retrieved data from a representative US population sample of 6,536 respondents polled in 2002 (August 19-September 8), 2005 (May 18–22), 2007 (April 23-May 6), 2009 (September 9–15), and 2010 (April 15-May 5). Sample sizes for these years were 1,501, 1,001, 2,026, 1,006, and 1,002, respectively. The datasets and full documentation on data-collection procedures are openly accessible online from the Pew Research Center's Global Attitudes Project at http://www.pewglobal.org/category/datasets/.

### Materials

Life satisfaction was measured by the Pew Research Center using the “ladder of life” measure of global life satisfaction [41]. Specifically, early on in each of the Pew surveys, respondents were asked to “Imagine a ladder with steps numbered from 0 at the bottom to 10 at the top. Suppose the top of the ladder represents the best possible life for you; and the bottom, the worst possible life for you.” In the following order, they were asked to indicate the step of the ladder that they felt they personally stood on at the present time, the step they were on 5 years ago, and the step they thought they would be on in 5 years. The ladder of life measure [41] correlates highly with the widely used multiple-item Satisfaction With Life Scale [42] and is regarded as a measure of global life satisfaction.

Political affiliation was measured by asking respondents whether they considered themselves to be Republican, Democrat or Independent (dummy coded 1, −1, and 0, respectively). Political orientation was measured by asking respondents whether they would describe their political views as “very conservative” (−2), “conservative” (−1), “moderate” (0), “liberal” (1), or “very liberal” (2).

As noted earlier, present political climate was measured as follows: if the president in power at the time of the survey shared the respondent's political affiliation (i.e., both Democrats or both Republicans), the political climate was favorable. If the president was not aligned with the respondent's political affiliation, (i.e., one Democratic and the other Republican), the political climate was unfavorable. Independents were always treated as being in an intermediate political climate. Thus, in 2002, 2005, and 2007, where the administration was Republican (with George W. Bush as president), the political climate was favorable for Republicans and unfavorable for Democrats. In contrast, in 2009 and 2010, where the administration was Democrat (with Barack Obama as president), the political climate was favorable for Democrats and unfavorable for Republicans. For analyses of past life satisfaction, we also examined past political climate. This variable was defined in the same way, except that it was based on the president in office 5 years before the survey was taken. Thus, for the 2002 data set, the past president was a Democrat (William J. Clinton in 1997) and, for all other years, the past president was a Republican (George W. Bush, from his incumbency in 2000 to his second term in office in 2005).

### Analysis

Variations in sample size across reported analyses reflect the effect of case-wise or list-wise deletion of cases with missing data. Analyses of covariance (ANCOVAs) were used to test the favorability and asymmetric sensitivity hypotheses. All ANCOVAs controlled for demographic factors typically controlled in other studies. These included respondents' sex (1 = male, 2 = female), age (years) and age squared (as in [4]), education level (1 = no high school, 2 = some high school, 3 = high school graduate, 4 = high school graduate plus vocational training, 5 = some college, 6 = college graduate, 7 = postgraduate or professional training after completing college), income (1 = <$10,000, 2 = $10,000–$20,000, 3 = $20,000–$30,000, 4 = $30,000–$40,000, 5 = $40,000–$50,000, 6 = $50,000–$75,000, 7 = $75,000–$100,000, 8 = >100,000), and relationship status (0 = never married, separated, divorced, or widowed; 1 = married or living with a partner).

Additionally, we controlled for religiosity (“How important is religion in your life?”; 1 = very important, 4 = not at all important), which has been shown to mediate the predictive effect of conservatism on life satisfaction [5]. We also controlled for US real (i.e., inflation-adjusted) gross domestic product (GDP) per capita as an exogenous, year-level indicator of economic conditions. Although we already control for personal income, we included real GDP per capita given that the years of favorable political climate for Republicans were prior to the Great Recession (i.e., 2002, 2005, and 2007), while those for Democrats were after the Great Recession (i.e., 2009 and 2010). Thus, we wanted to control for macro-level annual variations in an inflation-adjusted per capita economic indicator that is well regarded as a measure of a country's standard of living in a given year. Finally, ANCOVAs for future and past life satisfaction controlled for present life satisfaction, given that the latter was positively correlated with both past (*r* = .27, *p*<.001) and future (*r* = .50, *p*<.001) life satisfaction and assessed immediately prior to past and future assessments. Past and future life satisfaction measures were uncorrelated (r = −.02, *p* = .12) and ANCOVAs on each of these measures did not control for the other measure.

In addition to the ANCOVAs, we report comparable results from analyses of variance (ANOVAs) in which none of the aforementioned covariates were controlled, following the recommendations of [43]. Unless otherwise indicated, two-tailed significance values are reported and *p* values less than .05 are reported as significant. We report partial eta squared values as a measure of effect size for ANCOVA and ANOVA results. Although it has been proposed that omega squared is an unbiased measure of population effect size [44], it is now generally regarded that omega squared is systematically biased in that it underestimates effect size [45].

## Results

### Political Orientation of Political Affiliates

We first explored the asymmetric political extremity hypothesis. As expected, political orientation varied by political affiliation, *F*(2, 5881) = 509.06, *p*<.001, η_p_^2^ = .15, with all pair-wise groups differing significantly based on Tamahane's T2 post-hoc tests. On average, Democrats were slightly left of center (*N* = 2,090, *M* = −0.11, *SD* = 0.97), Independents were close to their mirror image, being slightly right of center (*N* = 1,957, *M* = 0.16, *SD* = 0.88), whereas Republicans were much farther to the right (*N* = 1,837, *M* = 0.78, *SD* = 0.79). In support of the asymmetric political extremity hypothesis, Republicans were substantially more polarized in their political orientation than Democrats. This was confirmed by comparing the absolute values of mean political orientation for Democrats and Republicans, which showed a large and significant effect, *t*′(3896.64) = 24.01, *p*<.001, Cohen's *d* = 0.76. In fact, Democrats were even less polarized than Independents, although the effect was very small and not significant, *t*′(4040.55) = 1.76, *p* = .079, Cohen's *d* = 0.06. The conservative-leaning mean political orientation of Independents is consistent with the Republican bias observed in their presidential voting behavior [15].

### Life Satisfaction

Table 1 shows the results of the fixed factors from four separate two-way (political affiliation × political climate) ANCOVAs that controlled the covariates described in the Method section. The four sets of results are for present life satisfaction, future life satisfaction, past life satisfaction with present political climate as a fixed factor, and past life satisfaction with past political climate as a fixed factor, respectively. Tables S1–S4 include a fuller description of the ANCOVAs summarized in Table 1 by including the results for each covariate. For each set of results, Table 1 also reports two simple effects that are pertinent to testing the asymmetric sensitivity hypothesis. Table 2 shows the comparable findings from four separate ANOVAs in which no covariate was controlled. Table 3 shows the descriptive findings and pairwise test results for main effects across the four sets of analyses that adjusted for the covariates, whereas Table 4 presents the comparable statistics when no covariates were included.

**Table 1:** Inferential tests of life satisfaction measures by political affiliation and political climate (adjusted for covariates).

DV	Model term	*F*	*df*	*p*	η_p_^2^
Present	PA	2.06	1, 5283	.151	.000
	PC	21.36	1, 5283	.000	.004
	PA×PC	2.96	1, 5283	.085	.001
	PC|PA = Dem	3.78	1, 1888	.052	.002
	PC|PA = Rep	20.16	1, 1614	.000	.012
Future	PA	7.68	1, 4965	.006	.002
	PC	30.43	1, 4965	.000	.006
	PA×PC	6.61	1, 4965	.010	.001
	PC|PA = Dem	5.46	1, 1761	.020	.003
	PC|PA = Rep	35.77	1, 1525	.000	.023
Past	PA	0.00	1, 5236	.997	.000
	PC	2.65	1, 5236	.104	.001
	PA×PC	7.55	1, 5236	.006	.001
	PC|PA = Dem	0.66	1, 1866	.417	.000
	PC|PA = Rep	14.19	1, 1599	.000	.009
Past	PA	1.43	1, 5236	.232	.000
	PC*	5.64	1, 5236	.018	.001
	PA×PC*	3.76	1, 5236	.053	.001
	PC*|PA = Dem	0.00	1, 1866	.997	.000
	PC*|PA = Rep	21.07	1, 1599	.000	.013

*Note*. DV  =  dependent variable, PA  =  political affiliation, and PC  =  present political climate, except for the last 4 rows where PC refers to past political climate and the levels are followed by an asterisk. Dem  =  Democrat, Rep  =  Republican.

**Table 2:** Inferential tests of life satisfaction measures by political affiliation and political climate (without covariates).

DV	Model term	*F*	*df*	*p*	η_p_^2^
Present	PA	21.78	1, 5998	.000	.004
	PC	25.73	1, 5998	.000	.004
	PA×PC	1.47	1, 5998	.226	.000
	PC|PA = Dem	7.81	1, 2128	.005	.004
	PC|PA = Rep	20.47	1, 1848	.000	.011
Future	PA	0.17	1, 5610	.682	.000
	PC	64.36	1, 5610	.000	.011
	PA×PC	10.17	1, 5610	.001	.002
	PC|PA = Dem	11.77	1, 1973	.001	.006
	PC|PA = Rep	66.87	1, 1735	.000	.037
Past	PA	5.97	1, 5958	.015	.001
	PC	1.38	1, 5958	.241	.000
	PA×PC	9.97	1, 5958	.002	.002
	PC|PA = Dem	2.12	1, 2107	.145	.001
	PC|PA = Rep	9.06	1, 1839	.003	.005
Past	PA	3.28	1, 5958	.070	.001
	PC*	4.92	1, 5958	.027	.001
	PA×PC*	15.51	1, 5958	.000	.003
	PC*|PA = Dem	1.54	1, 2107	.219	.001
	PC*|PA = Rep	19.19	1, 1839	.000	.010

*Note*. DV  =  dependent variable, PA  =  political affiliation, and PC  =  present political climate, except for the last 4 rows where PC refers to past political climate and the levels are followed by an asterisk. Dem  =  Democrat, Rep  =  Republican.

**Table 3:** Life satisfaction measures bypolitical affiliation and present political climate (adjusted for covariates).

		Political affiliation							
		Democrat		Independent		Republican		Average	
DV	PC	Mean	SE	Mean	SE	Mean	SE	Mean	SE
Present	Fav.	7.03	0.08			7.24	0.05	7.14^a^	0.05
	Int.			6.92	0.04			6.92^b^	0.04
	Unfav.	6.83	0.05			6.81	0.09	6.82^b^	0.05
	Avg.	6.93^a^	0.05	6.92^a^	0.04	7.03^a^	0.05		
Future	Fav.	8.14	0.08			8.13	0.06	8.13^a^	0.05
	Int.			7.85	0.04			7.85^b^	0.04
	Unfav.	7.93	0.05			7.57	0.09	7.76^b^	0.05
	Avg.	8.04^a^	0.05	7.85^b^	0.04	7.85^b^	0.05		
Past	Fav.	6.43	0.09			6.20	0.07	6.31^a^	0.06
	Int.			6.40	0.05			6.40^a^	0.05
	Unfav.	6.34	0.06			6.56	0.10	6.45^a^	0.06
	Avg.	6.38^a^	0.06	6.40^a^	0.05	6.38^a^	0.06		
Past	Fav.	6.37	0.09			6.45	0.08	6.41^a^	0.06
	Int.			6.40	0.05			6.40^a^	0.05
	Unfav.	6.36	0.07			6.09	0.09	6.23^b^	0.05
	Avg.	6.36^a^	0.05	6.40^a^	0.05	6.27^a^	0.06		

*Note*. DV  = dependent variable. PC  =  present political climate, except for thelast 4 rows where PC refers to past political climate. Fav.  =  favorable,Int.  =  intermediate, Unfav.  =  unfavorable, and Avg.  = average. Means are estimated marginal means from the associated ANCOVA models (Table?1). Superscripted letters that differ withinthe “Average” rows or columns (within a level of DV)denote means that differ significantly at *p*<.05based on 1,000 bootstrap samples.

**Table 4:** Life satisfaction measures by political affiliation and present political climate (without covariates).

		Political affiliation							
		Democrat		Independent		Republican		Average	
DV	PC	Mean	SE	Mean	SE	Mean	SE	Mean	SE
Present	Fav.	7.01	0.08			7.40	0.05	7.21^a^	0.05
	Int.			6.92	0.04			6.92^b^	0.04
	Unfav.	6.75	0.05			6.98	0.09	6.86^b^	0.05
	Avg.	6.88^a^	0.05	6.92^a^	0.04	7.19^b^	0.05		
Future	Fav.	8.14	0.09			8.36	0.06	8.25^a^	0.06
	Int.			7.82	0.05			7.82^b^	0.05
	Unfav.	7.74	0.06			7.44	0.10	7.59^c^	0.06
	Avg.	7.94^a^	0.06	7.82^a^	0.05	7.90^a^	0.06		
Past	Fav.	6.47	0.09			6.41	0.07	6.44^ab^	0.06
	Int.			6.37	0.05			6.37^a^	0.05
	Unfav.	6.31	0.06			6.77	0.10	6.54^b^	0.06
	Avg.	6.39^a^	0.06	6.37^a^	0.05	6.59^b^	0.06		
Past	Fav.	6.27	0.08			6.71	0.07	6.49^a^	0.06
	Int.			6.37	0.05			6.37^ab^	0.05
	Unfav.	6.40	0.06			6.24	0.08	6.32^b^	0.05
	Avg.	6.34^a^	0.05	6.37^a^	0.05	6.48^a^	0.06		

*Note*. DV  =  dependent variable. PC  =  present political climate, except for the last 4 rows where PC refers to past political climate. Fav.  =  favorable, Int.  =  intermediate, Unfav.  =  unfavorable, and Avg.  =  average. Means are estimated marginal means from the associated ANOVA models (Table 2). Superscripted letters that differ within the “Average” rows or columns (within a level of DV) denote means that differ significantly at *p*<.05 based on 1,000 bootstrap samples.

#### Present life satisfaction

With the covariates controlled, the main effect of political affiliation on present life satisfaction was not significant (Table 1 and Table S1). However, a very small, significant effect of political affiliation was found without controlling the covariates (Table 2). In that analysis, Republicans were significantly more satisfied than Democrats and Independents (Table 4).

In support of the favorability hypothesis, there was a very small, significant effect of political climate such that respondents in a favorable climate were more satisfied with their lives than respondents in an unfavorable climate, both when covariates were controlled (Tables 1 and S1) and when they were not (Table 2). Moreover, Independents' mean life satisfaction fell between the means for the favorable and unfavorable political-climate groups, being significantly lower than the favorable group and not significantly different from the unfavorable group (Tables 3 and 4). These hypotheses can be jointly tested by means of an ordered heterogeneity test [46]. Let *r*_s_ *P*_c_ equal the product of the Spearman correlation, *r*_s_, between the rank order of the observed and predicted group means and the complement of the two-tailed probability of the political climate main effect. The ordered heterogeneity test scales support for order hypotheses between 0 (no support) and 1 (strongest support), and [46] provides significance tables for the statistic. In the present case, *r*_s_ = 1, and *P*_c_ = .999. Thus, *r*_s_ *P*_c_ = .999, *p*<.001, yielding very strong support for the ordered (favorability) hypothesis.

The asymmetric sensitivity hypothesis, however, was not supported in our initial test. The political affiliation by political climate interaction effect did not reach significance regardless of whether the covariates were controlled (Table 1 and Table S1) or not (Table 2). Although the interaction was nonsignificant, the simple effect of political climate is larger for Republicans than for Democrats (Table 1). We note this because it affects the sign of the *F* test used in our subsequent meta-analysis (i.e., although the *F* value is nonsignificant, it is positive).

#### Future life satisfaction

With covariates controlled, there was a very small, significant main effect of political affiliation on future life satisfaction (Table 1 and Table S2). Democrats were significantly more optimistic about their future life satisfaction than Republicans (Table 3). Democrats were also more optimistic about future satisfaction than Independents, who did not significantly differ from Republicans (Table 3). However, the main effect was not significant when the covariates were not controlled (Table 2), casting some uncertainty on the basis of the controlled effect.

Supporting the favorability hypothesis, there was a very small, significant main effect of political climate showing that respondents in a favorable political climate were more optimistic than respondents in an unfavorable climate, both when covariates were controlled (Table 1 and Table S2) and when they were not (Table 2). Once again, Independents' mean satisfaction fell between the favorable and unfavorable political-climate group means (cf. Tables 3 and 4). An ordered heterogeneity test showed very strong support for the favorability hypothesis and its corollary prediction of nonpartisan intermediacy, *r*_s_ *P*_c_ = .999, *p*<.001.

Supporting the asymmetric sensitivity hypothesis, a very small, significant interaction effect was found, both when the covariates were controlled (Table 1 and Table S2) and not controlled (Table 2). As Tables 1 and 2 also show, the effect of political climate on anticipated future life satisfaction was stronger among Republicans than among Democrats.

#### Past life satisfaction

Past life satisfaction did not differ by political affiliation when covariates were controlled (Table 1, Tables S3 and S4). Without the covariates, the main effect was significant in one analysis (with present political climate as a fixed factor) and nonsignificant in the other (with past political climate as a fixed factor) (Table 2). Taken together, these findings indicate that past life satisfaction is not robustly influenced by political affiliation. Even in the one analysis where the effect reached significance, it was very small.

The main effect of present political climate on past life satisfaction was nonsignificant (Tables 1, 2, and Table S3). Thus, we did not find overall support for the contrast effect proposed on the basis of the inclusion/exclusion model. However, consistent with the assimilation effect predicted on the basis of the same model, there was a very small but significant main effect of past political climate, both when the covariates were controlled (Table 1, Table S4) and when they were not controlled (Table 2). Respondents in a favorable past political climate recalled being significantly more satisfied with their lives in the past than those in an unfavorable past climate (Tables 3 and 4). Independents were intermediate and the ordered heterogeneity test was significant, *r*_s_ *P*_c_ = .882, *p*<.025.

The interaction effect between political affiliation and present political climate was very small but significant, both controlling for covariates (Table 1 and Table S3) and without covariates (Table 2). Consistent with the predicted contrast effect, Republicans recalled being more satisfied in the past when the present climate was unfavorable, but not so for Democrats who showed no contrast effect (Tables 3 and 4). Consistent with the predicted assimilation effect, Republicans recalled being more satisfied in the past when the past climate was favorable, but not so for Democrats who showed no assimilation effect (Tables 3 and 4). Thus, compared to Democrats, Republicans were more strongly affected by both the present and past political climates in the manner predicted by the inclusion/exclusion model.

### Meta-analyses

Given that we had four tests of the favorability and asymmetric sensitivity hypotheses, we meta-analyzed the four significance test results per hypothesis. We did this separately for tests that used the covariates and for those that did not. Following Equation 19 in [47], exact one-tailed probabilities of the four political climate main effects and the political affiliation by political climate interaction effects were derived from the *F* scores shown in Tables 1 and 2 and then converted to *Z* scores (Table S5). The sum of the four *Z* scores per meta-analytic test was divided by the square root of 4 (i.e., the number of tests).

Regarding the favorability hypothesis, the combined effect of political climate was highly significant when the covariates were controlled (*Z* = 6.68, *p*<.0000001) and when the covariates were not controlled (*Z* = 6.45, *p*<.0000001). Regarding the asymmetric sensitivity hypothesis, the combined interaction effect was highly significant with the covariates controlled (*Z* = 4.49, *p* = .000004) and with the covariates not controlled (*Z* = 5.74, *p*<.0000001). These findings unambiguously support both the favorability and asymmetric sensitivity hypotheses, bearing in mind that the effects reported in this study are also invariably very small even where significant, a point we address towards the end of the Discussion.

## Discussion

Our findings support the view that political group differences in life satisfaction are best understood within a P×S interactionist framework. We interpret the differential sensitivity of Republicans and Democrats to political climate in terms of a reciprocal and subjectivist form of interactionism, congruent with cognitive-affective systems theory [13]. In this conceptualization, political climate is a situational factor (“Who is the president in power?”) that is meaningfully encoded by individuals (“Is the president aligned with my political group membership?”). Political climate may activate and influence cognitive and affective reactions in those individuals (such as their evaluations of life satisfaction), which may also be moderated by individual or group differences, such as party affiliation.

We expressed these ideas in terms of three hypotheses. In support of the first, we found evidence of a *favorability effect*: partisans were more satisfied with their current lives when the political climate was favorable to them than when it was unfavorable. They were more optimistic about their future life satisfaction when the present political climate was favorable rather than unfavorable. And, they recalled being more satisfied with their lives five years earlier when, at that time, the political climate was favorable to them rather than when it was not. Finally, as we also expected, nonpartisan Independents expressed levels of satisfaction that fell between those who experienced favorable or unfavorable climates. The favorability effect is consistent with other findings indicating that partisans' assessments on a range of topics are influenced by the interaction of political conditions and their political views. For instance, US partisans' views of the president have a much stronger effect on their views of the economy than the other way around [21]. Our findings also corroborate the finding of an earlier study showing that partisans were happier after experiencing a Congressional victory rather than a Congressional loss [23]. Unlike that study, however, this research did not employ a pre-post panel design, thus ruling out the possibility that such effects were due to inadvertent, yet not improbable, experimental cuing.

Just as the favorability hypothesis points to the limits of dispositional accounts of liberal-conservative differences by showing how political climate captures important P×S effects, likewise the asymmetric sensitivity hypothesis (the second of our three core hypotheses) point to the limits of such “congruence-based” predictions when left unqualified by political group differences. Our findings showed that Republicans are more sensitive than Democrats to the effect of their present political climate on past and future life satisfaction (the difference for present life satisfaction, while in the predicted direction, was not significant). Republicans were also more sensitive than Democrats to the political climate that existed 5 years earlier in assessing how satisfied with their lives they were at that time.

Indeed, asymmetric sensitivity effects on past life satisfaction revealed that, on average, whereas Democrats showed no sign of assimilation to the past political climate or contrast from the present political climate, Republicans exhibited both of these effects. That is, Republicans' retrospective assessments of life satisfaction were assimilated to political conditions that existed at the recalled time of experience 5 years prior to evaluation, and those same assessments were contrasted away from the political conditions that existed in different political times; namely, at the later time of evaluation. This suggests that not only are Republicans more sensitive than Democrats to the present political climate, they are also affected by political climate in more complex ways that can involve multiple temporal reference points with opposing effects, such as the joint pattern of assimilation and contrast seen in this study. As noted earlier, the inclusion/exclusion model [38] can account for this pattern of finding. However, that model neither predicts how group differences (political or otherwise) might moderate the fit of the proposed social-cognitive processes nor has it been applied to this sort of prediction. Our research therefore shows how the inclusion/exclusion model might be generalized to other evaluative contexts and how its predictions may be qualified by predictable group differences.

The asymmetric sensitivity effects shown in this research were predicated on yet another hypothesized political group asymmetry (our third hypothesis) that was also confirmed: the *asymmetric political extremity effect*. We showed that Republicans located themselves much farther to the right of center than Democrats located themselves to the left of center on a standard political orientation measure. It would be of value to examine in future research how Democrats and Republicans rate themselves on a bi-dimensional political orientation scale that includes separate measures of liberalism and conservatism [35]. Regardless of whether conservatives have a predilection for value-conflict minimization [33] or whether they simply weight conflicting values less equally than liberals [48], we expect that Democrats would score higher on conservatism than Republicans would score on liberalism. However, if Republicans not only have less value-conflicted political identities than Democrats, but also stronger core political identities [29], Republicans might also be expected to score higher on conservatism than Democrats to score on liberalism. Future research could assess the contribution of these two possible sources of the asymmetric political extremity effect.

Our findings also revealed that political group differences in life satisfaction are not consistent across past, present, and future timeframes and depend on whether or not covariates are statistically controlled. First, we found that the “happiness boost” for Republicans reported in earlier work [4]–[6] was only replicated without the use of statistical controls, as [9] also found. Even so, the effect was very small. This finding may seem at striking odds with past research, but the inconsistency is more apparent than real if we consider the effect sizes and methodological specification in earlier research. Although [4] did not report effect sizes or statistics that would allow for effect size calculations and [6] reports only descriptive results, [5] reported effect sizes. In Study 1 of [5], the unmediated correlation between political orientation and life satisfaction was between small and medium (*r* = .18). Yet [5] did not clearly specify whether political orientation was measured before or after happiness. However, judging by the order in which the measures are mentioned in [5], it appears that political orientation was measured directly before happiness (see footnote 1 in [5]). In Study 2 [5], which utilized data from 3,692 respondents of the World Values Survey, the correlation between conservatism and life satisfaction (with no covariates) was .13. Not only is this effect small, the World Values Survey asks respondents about the effect of politics on their life just a few questions before eliciting their life satisfaction. Thus, the test is much less conservative than ours. Finally, in Study 3 [5], which utilized GSS data from 41,719 respondents ([4] also drew on this data set), the correlation between conservatism and happiness (again without covariates) was .07. As we noted earlier, the GSS queries respondents about their happiness after eliciting political measures. In [9], there was a small effect in which conservatism correlated .08 with life satisfaction. Once again, the method section in [9] does not clearly specify the order in which measures were taken, although the life satisfaction measure is described after the political orientation measures, suggesting that the order of elicitation corresponded to the order of description. In short, the focus on significance testing in past research, coupled with large samples and more liberal research designs, may have contributed to the perception that the happiness gap is larger than it is.

In terms of anticipated future life satisfaction, no significant effect of political affiliation was found when covariates were not included, but Democrats were found to be more optimistic about their future life satisfaction than Republicans when the covariates were controlled. One important difference between the control procedures in this case and the earlier one is that the ANCOVA of future life satisfaction controlled for present life satisfaction, whereas the ANCOVA of present life satisfaction did not control for future life satisfaction. However, we verified that even with present life satisfaction removed as a covariate, the effect of political affiliation of future life satisfaction was nonsignificant. Moreover, we found virtually no difference in recollected life satisfaction 5 years earlier between Democrats, Independents, and Republicans. Taken together, the present findings show that group-level effects of political affiliation on life satisfaction measures are very small at best. Such findings pose an explanatory challenge for person- or group-level accounts that would seem to predict larger effects [4], [5], [9].

### Directions for Future Research

The present research offers several directions for future work, of which we mention a few. Future research could address the generalizability of the present findings over a longer timeframe in which different Republican and Democratic presidents are in power. In our study, the Republican period covered the Bush years 2002, 2005, and 2007 and the Democratic period covered the Obama years of 2009 and 2010 (as well as the Clinton year of 1997 the case of past political climate). It would be of value to examine how Republicans and Democrats respond to future Democratic and Republican administrations using methods that elicit measures of subjective well-being prior to questioning respondents about their political affiliations and views. In particular, a concern of ours was that the Bush years preceded the Great Recession and the Obama years followed it. Thus, differences attributable to political climate may have been due to changing economic conditions. Our findings, however, do not support this explanation. First, we controlled for both personal income and real GDP per capita. Second, although income was a significant covariate of past and present life satisfaction, it was not a significant covariate of future life satisfaction. Yet, our analyses of future life satisfaction revealed strong support for both the favorability and asymmetric sensitivity hypotheses. Finally, real GDP per capita was not a significant covariate in any analysis, which is consistent with recent research showing that the relationship between life satisfaction and per capita GDP is relatively flat in richer countries [49].

Future research could also explore moderators of the effect sizes observed in this study. As we noted repeatedly, the effects reported in this study were, almost without exception, very small. The one exception was the large difference in political orientation extremity between Democrats and Republicans. Although our meta-analyses revealed highly significant combined effects supporting the favorability hypothesis and the asymmetric sensitivity hypothesis, those effects were invariably very small. As we noted at the outset, this is unsurprising given the research methods employed by the Pew Research Center. Recall that the life satisfaction questions were posed to respondents at the start of the survey. Respondents were not primed with any politically relevant cues prior to answering these questions. Thus, the observed effects of political affiliation, political climate, and their interaction were unobtrusive and distanced from one another. We anticipate consistent but larger effects to be seen in experimental designs in which Democrats and Republicans are presented with political group-relevant information that is either affectively positive or negative before rating their life satisfaction. We predict that, under such conditions, the effect of climate (defined in terms of the valence of information received) and the climate by political affiliation interaction effect would be closer to a medium effect size.

It would also be instructive in future research to examine how partisans' present and future life satisfaction might be influenced by expectations regarding the *future* political climate or by contemplation of a particular future scenario that is either favorable or unfavorable. We expect that such a test would further generalize support for the asymmetric sensitivity hypothesis. For instance, we predict that if Republicans and Democrats were randomly assigned to conditions in which they either imagined a future political win or a future political loss for their party in the next presidential election, the effect on their assessments of present life satisfaction and on their expectations about future life satisfaction would be greater for Republicans than for Democrats.

Finally, we note that the asymmetric sensitivity hypothesis draws an intriguing connection to recent work on power and subjective well-being. For instance, it has been shown that participants' subjective power predicts their subjective well-being, and this effect is mediated by feelings of authenticity [50]. Our findings suggest that political affiliation might serve as an important moderator of this mediated effect. That is, we expect that the mediated effect would be stronger among Republicans than among Democrats. When Republicans have their party in power, they show evidence of heightened subjective well-being compared to when their party is not in power. In contrast, Democrats showed a more “even keel” response to changing political climates, which may indicate that their subjective well-being is less responsive to fluctuating levels of power or that their sense of power is less closely tied to the political climate than it is for Republicans. Given that Democrats' political objectives tend to focus on social change, while Republicans' political objectives tend to focus on social conservation, one possibility is that even when Democrats have their party in power, their relative sense of power may be weak because their transformative objectives are often more difficult to achieve and yield progress at a slower pace than Republicans' conservative objectives. Moreover, although *having* power increases subjective well-being, *striving* for power may decrease it [50]. Thus, the liberal sense of striving for change might undermine the effect of Democrats' political power on their subjective well-being. These hypotheses could be tested in future research.

## Conclusions

The present research illustrates the value of Person × Situation interactionism in examining political group differences in life satisfaction. Not only does life satisfaction predictably vary by political climate, such that people tend to be more satisfied when their party is in power than when it is not, the effect of political climate clearly differs by political group membership. Compared to Democrats, Republicans were more sensitive to political climate. Republicans were also more politically polarized. Perhaps Thomas Nast was onto something when he chose to characterize Republicans as elephants and Democrats as donkeys, as our findings do seem to suggest that Republicans are bigger political animals.

## Supporting Information

Table S1 **Inferential tests of present life satisfaction by political affiliation and present political climate.**
(DOCX)

Table S2 **Inferential tests of future life satisfaction by political affiliation and present political climate.**
(DOCX)

Table S3 **Inferential tests of past life satisfaction by political affiliation and present political climate.**
(DOCX)

Table S4 **Inferential tests of past life satisfaction by political affiliation and past political climate.**
(DOCX)

Table S5 ***Z*** **scores for meta-analysis of combined significance tests.**
(DOCX)

## References

[1] Diener E, Suh EM, Lucas RE, Smith HL (1999) Subjective well-being: Three decades of progress. Psychol Bull 125: 276.

[2] Seligman ME, Csikszentmihalyi M (2000) Positive psychology: An introduction. Am Psychol 55: 5. 11392865 10.1037//0003-066x.55.1.5

[3] Carroll J (2007) Most Americans very satisfied with their personal lives. Gallup report. Available: http://www.gallup.com/poll/103483/most-americans-very-satisfiedtheir-personal-lives.aspx. Accessed 2013 Dec 26.

[4] Napier JL, Jost JT (2008) Why are conservatives happier than liberals? Psychol Sci 19: 565–572. 18578846 10.1111/j.1467-9280.2008.02124.x

[5] Schlenker BR, Chambers JR, Le BM (2012) Conservatives are happier than liberals, but why? Political ideology, personality, and life satisfaction. J Res Pers 46: 127–146.

[6] Taylor P, Funk C, Craighill P (2006) Are we happy yet? Pew Research Center. Available: http://www.pewsocialtrends.org/2006/02/13/are-we-happy-yet/. Accessed 2013 Dec 26.

[7] Jost JT, Hunyady O (2005) Antecedents and consequences of system-justifying ideologies. Curr Dir Psychol Sci 14: 260–265.

[8] Vigil JM (2010) Political leanings vary with facial expression processing and psychosocial functioning. Group Process Intergroup Relat 13: 547–558.

[9] Jetten J, Haslam SA, Barlow FK (2013) Bringing back the system one reason why conservatives are happier than liberals is that higher socioeconomic status gives them access to more group memberships. Soc Psychol Personal Sci 4: 6–13.

[10] Myers DG (2000) The funds, friends, and faith of happy people. Am Psychol 55: 56. 11392866 10.1037//0003-066x.55.1.56

[11] Heller D, Watson D, Ilies R (2004) The role of person versus situation in life satisfaction: A critical examination. Psychol Bull 130: 574. 15250814 10.1037/0033-2909.130.4.574

[12] Endler NS, Magnusson D (1976) Toward an interactional psychology of personality. Psychol Bull 83: 956. 794903

[13] Mischel W, Shoda Y (1995) A cognitive-affective system theory of personality: Reconceptualizing situations, dispositions, dynamics, and invariance in personality structure. Psychol Rev 102: 246. 7740090 10.1037/0033-295x.102.2.246

[14] Ross L, Nisbett RE (1991) The person and the situation: Perspectives of social psychology. New York: McGraw-Hill Book Company.

[15] Bartels LM (2000) Partisanship and voting behavior, 1952–1996. Am J Polit Sci 44: 35–50.

[16] Dodd MD, Balzer A, Jacobs CM, Gruszczynski MW, Smith KB, et al (2012) The political left rolls with the good and the political right confronts the bad: Connecting physiology and cognition to preferences. Phil Trans R Soc B 367: 640–649. 22271780 10.1098/rstb.2011.0268 PMC3260844

[17] Schwarz N, Strack F (1999) Reports of subjective well-being: Judgmental processes and their methodological implications. In: Kahneman D, Diener E, Schwarz N. Well-being: The foundations of hedonic psychology. New York: Russell Sage Foundation. pp. 61–84.

[18] Krizan Z, Miller JC, Johar O (2010) Wishful thinking in the 2008 US presidential election. Psychol Sci 21: 140–146. 20424035 10.1177/0956797609356421

[19] Holbrook AL, Krosnick JA, Visser PS, Gardner WL, Cacioppo JT (2001) Attitudes toward presidential candidates and political parties: Initial optimism, inertial first impressions, and a focus on flaws. Am J Polit Sci: 930–950.

[20] Evans G, Andersen R (2006) The political conditioning of economic perceptions. J Polit 68: 194–207.

[21] Evans G, Pickup M (2010) Reversing the causal arrow: The political conditioning of economic perceptions in the 2000–2004 US presidential election cycle. J Polit 72: 1236–1251.

[22] Johnston R, Sarker R, Jones K, Bolster A, Propper C, et al (2005) Egocentric economic voting and changes in party choice: Great Britain 1992–2001. Journal of Elections, Public Opinion & Parties 15: 129–144.

[23] Gerber AS, Huber GA (2010) Partisanship, political control, and economic assessments. Am J Polit Sci 54: 153–173.

[24] Inbar Y, Pizarro DA, Bloom P (2009) Conservatives are more easily disgusted than liberals. Cogn Emot 23: 714–725.

[25] Smith KB, Oxley D, Hibbing MV, Alford JR, Hibbing JR (2011) Disgust sensitivity and the neurophysiology of left-right political orientations. PLoS ONE 6: e25552. 22039415 10.1371/journal.pone.0025552 PMC3198440

[26] Carraro L, Castelli L, Macchiella C (2011) The automatic conservative: Ideology-based attentional asymmetries in the processing of valenced information. PLoS ONE 6: e26456. 22096486 10.1371/journal.pone.0026456 PMC3212508

[27] Oxley DR, Smith KB, Alford JR, Hibbing MV, Miller JL, et al (2008) Political attitudes vary with physiological traits. Sci 321: 1667–1670. 10.1126/science.1157627 18801995

[28] Joel S, Burton CM, Plaks JE (2014) Conservatives anticipate and experience stronger emotional reactions to negative outcomes. J Pers 82: 32–43. 23336710 10.1111/jopy.12031

[29] Graham J, Nosek BA, Haidt J (2012) The moral stereotypes of liberals and conservatives: Exaggeration of differences across the political spectrum. PLoS ONE 7: e50092. 23251357 10.1371/journal.pone.0050092 PMC3520939

[30] Janoff-Bulman R, Sheikh S, Baldacci KG (2008) Mapping moral motives: Approach, avoidance, and political orientation. J Exp Soc Psychol 44: 1091–1099.

[31] Amodio DM, Jost JT, Master SL, Yee CM (2007) Neurocognitive correlates of liberalism and conservatism. Nat Neurosci 10: 1246–1247. 17828253 10.1038/nn1979

[32] Critcher CR, Huber M, Ho AK, Koleva SP (2009) Political orientation and ideological inconsistencies:(dis) comfort with value tradeoffs. Soc Justice Res 22: 181–205.

[33] Jost JT, Glaser J, Kruglanski AW, Sulloway FJ (2003) Political conservatism as motivated social cognition. Psychol Bull 129: 339. 12784934 10.1037/0033-2909.129.3.339

[34] Nam HH, Jost JT, Van Bavel JJ (2013) “Not for all the tea in china!” political ideology and the avoidance of dissonance-arousing situations. PLoS ONE 8: e59837. 23620724 10.1371/journal.pone.0059837 PMC3631191

[35] Choma BL, Hafer CL, Dywan J, Segalowitz SJ, Busseri MA (2012) Political liberalism and political conservatism: Functionally independent? Pers Individ Dif 53: 431–436.

[36] Duckitt J, Sibley CG (2009) A dual-process motivational model of ideology, politics, and prejudice. Psychol Inq 20: 98–109.

[37] Bonaparte Y, Kumar A, Page J (2012) Political climate, optimism, and investment decisions. AFA 2012 Chicago Meetings Paper. Available SSRN: http://ssrn.com/abstract=1509168 or http://dx.doi.org/10.2139/ssrn.1509168

[38] Bless H, Schwarz N (2010) Mental construal and the emergence of assimilation and contrast effects: The inclusion/exclusion model. Adv Exp Soc Psychol 42: 319–373.

[39] Cohen J (1988) Statistical power analysis for the behavioral sciences (2^nd^ ed.). Hillsdale, NJ: Erlbaum.

[40] Glass GV, McGaw B, Smith ML (1981) Meta-analysis in social research. Beverly Hills, CA: Sage.

[41] Cantril H (1965) The patterns of human concern. New Brunswick, NJ: Rutgers University Press.

[42] Diener E, Emmons RA, Larsen RJ, Griffin S (1985) The satisfaction with life scale. J Pers Assess 49: 71–75. 16367493 10.1207/s15327752jpa4901_13

[43] Simmons JP, Nelson LD, Simonsohn U (2011) False-positive psychology: undisclosed flexibility in data collection and analysis allows presenting anything to seem significant. Psychol Sci 22: 1359–1366. 22006061 10.1177/0956797611417632

[44] Hays WL (1963) Statistics. New York: Holt, Rinehart, & Winston.

[45] Richardson JTE (2011) Eta squared and partial eta squared as measures of effect size in educational research. Educational Research Review 6: 135–147.

[46] Rice WR, Gaines SD (1994) The ordered-heterogeneity family of tests. Biom 50: 746–752.

[47] Rosenthal R (1991) Meta-analysis: A review. Psychosom Med 53: 247–271. 1882008 10.1097/00006842-199105000-00001

[48] Tetlock PE (1983) Cognitive style and political ideology. J Pers Soc Psychol 45: 118.

[49] Proto E, Rustichini A (2013) A reassessment of the relationship between GDP and life satisfaction. PLoS ONE 8: e79358. 24312179 10.1371/journal.pone.0079358 PMC3842267

[50] Kifer Y, Heller D, Perunovic WQE, Galinsky AD (2013) The good life of the powerful the experience of power and authenticity enhances subjective well-being. Psychol Sci 24: 280–288. 23322701 10.1177/0956797612450891

