# Supplementary material for: Political Differences in Past, Present, and Future Life Satisfaction: Republicans Are More Sensitive than Democrats to Political Climate
Source: PLoS One. 2014 Jun 5;9(6):e98854. doi: 10.1371/journal.pone.0098854 (PMC4047066; doi:10.1371/journal.pone.0098854)
Supplement: Table S1 — Inferential tests of present life satisfaction by political affiliation and present political climate. (DOCX) [file pone.0098854.s001.docx]

**Table S1. Inferential tests of present life satisfaction by political affiliation and present political climate.**

| Model term | *F* | *df* | *p* | η_p_^2^ |
| --- | --- | --- | --- | --- |
| Sex | 24.61 | 1, 5283 | .000 | .005 |
| Age | 27.99 | 1, 5283 | .000 | .005 |
| Age-squared | 38.71 | 1, 5283 | .000 | .007 |
| Relationship | 37.14 | 1, 5283 | .000 | .007 |
| Education | 14.12 | 1, 5283 | .000 | .003 |
| Income | 192.09 | 1, 5283 | .000 | .035 |
| Religiosity | 12.96 | 1, 5283 | .000 | .002 |
| Real GDP per cap. | 1.29 | 1, 5283 | .255 | .000 |
| PA | 2.06 | 1, 5283 | .151 | .000 |
| PC | 21.36 | 1, 5283 | .000 | .004 |
| PA×PC | 2.96 | 1, 5283 | .085 | .001 |
| PC\|PA=Dem | 3.78 | 1, 1888 | .052 | .002 |
| PC\|PA=Rep | 20.16 | 1, 1614 | .000 | .012 |

*Note*. PA = political affiliation, PC = present political climate (i.e., at time of polling), Dem = Democrat, Rep = Republican.
