# Supplementary material for: Political Differences in Past, Present, and Future Life Satisfaction: Republicans Are More Sensitive than Democrats to Political Climate
Source: PLoS One. 2014 Jun 5;9(6):e98854. doi: 10.1371/journal.pone.0098854 (PMC4047066; doi:10.1371/journal.pone.0098854)
Supplement: Table S2 — Inferential tests of future life satisfaction by political affiliation and present political climate. (DOCX) [file pone.0098854.s002.docx]

**Table S2. Inferential tests of future life satisfaction by political affiliation and present political climate.**

| Model term | *F* | *df* | *p* | η_p_^2^ |
| --- | --- | --- | --- | --- |
| Sex | 9.00 | 1, 4965 | .003 | .002 |
| Age | 9.51 | 1, 4965 | .002 | .002 |
| Age-squared | 6.73 | 1, 4965 | .010 | .001 |
| Relationship | 6.42 | 1, 4965 | .011 | .001 |
| Education | 8.43 | 1, 4965 | .004 | .002 |
| Income | 0.28 | 1, 4965 | .595 | .000 |
| Religiosity | 1.39 | 1, 4965 | .238 | .000 |
| Real GDP per cap. | 1.59 | 1, 4965 | .208 | .000 |
| Present satisfaction | 1903.14 | 1, 4965 | .000 | .277 |
| PA | 7.68 | 1, 4965 | .006 | .002 |
| PC | 30.43 | 1, 4965 | .000 | .006 |
| PA×PC | 6.61 | 1, 4965 | .010 | .001 |
| PC\|PA=Dem | 5.46 | 1, 1761 | .020 | .003 |
| PC\|PA=Rep | 35.77 | 1, 1525 | .000 | .023 |

*Note*. PA = political affiliation, PC = present political climate (i.e., at time of polling), Dem = Democrat, Rep = Republican.
