# Supplementary material for: Political Differences in Past, Present, and Future Life Satisfaction: Republicans Are More Sensitive than Democrats to Political Climate
Source: PLoS One. 2014 Jun 5;9(6):e98854. doi: 10.1371/journal.pone.0098854 (PMC4047066; doi:10.1371/journal.pone.0098854)
Supplement: Table S3 — Inferential tests of past life satisfaction by political affiliation and present political climate. (DOCX) [file pone.0098854.s003.docx]

**Table S3. Inferential tests of past life satisfaction by political affiliation and present political climate.**

| Model term | *F* | *df* | *p* | η_p_^2^ |
| --- | --- | --- | --- | --- |
| Sex | 0.01 | 1,5236 | .916 | .000 |
| Age | 0.08 | 1,5236 | .776 | .000 |
| Age-squared | 16.03 | 1,5236 | .000 | .003 |
| Relationship | 0.03 | 1,5236 | .853 | .000 |
| Education | 0.13 | 1,5236 | .721 | .000 |
| Income | 16.73 | 1,5236 | .000 | .003 |
| Religiosity | 9.35 | 1,5236 | .002 | .002 |
| Real GDP per cap. | 0.05 | 1,5236 | .828 | .000 |
| Present satisfaction | 291.51 | 1,5236 | .000 | .053 |
| PA | 0.00 | 1,5236 | .997 | .000 |
| PC | 2.65 | 1,5236 | .104 | .001 |
| PA×PC | 7.55 | 1,5236 | .006 | .001 |
| PC\|PA=Dem | 0.66 | 1,1866 | .417 | .000 |
| PC\|PA=Rep | 14.19 | 1,1599 | .000 | .009 |

*Note*. PA = political affiliation, PC = present political climate (i.e., at time of polling), Dem = Democrat, Rep = Republican.
