# Supplementary material for: Political Differences in Past, Present, and Future Life Satisfaction: Republicans Are More Sensitive than Democrats to Political Climate
Source: PLoS One. 2014 Jun 5;9(6):e98854. doi: 10.1371/journal.pone.0098854 (PMC4047066; doi:10.1371/journal.pone.0098854)
Supplement: Table S4 — Inferential tests of past life satisfaction by political affiliation and past political climate. (DOCX) [file pone.0098854.s004.docx]

**Table S4. Inferential tests of past life satisfaction by political affiliation and past political climate.**

| Model term | *F* | *df* | *p* (*p*_dir_) | η_p_^2^ |
| --- | --- | --- | --- | --- |
| Sex | 0.01 | 1,5236 | .930 | .000 |
| Age | .080 | 1,5236 | .777 | .000 |
| Age-squared | 16.04 | 1,5236 | .000 | .003 |
| Relationship | 0.12 | 1,5236 | .731 | .000 |
| Education | 0.16 | 1,5236 | .692 | .000 |
| Income | 17.49 | 1,5236 | .000 | .003 |
| Religiosity | 9.15 | 1,5236 | .003 | .002 |
| Real GDP per cap. | 0.77 | 1,5236 | .379 | .000 |
| Present satisfaction | 290.96 | 1,5236 | .000 | .053 |
| PA | 1.43 | 1,5236 | .232 | .000 |
| PC | 5.64 | 1,5236 | .018 | .001 |
| PA×PC | 3.76 | 1,5236 | .053 | .001 |
| PC\|PA=Dem | 0.00 | 1,1866 | .997 | .000 |
| PC\|PA=Rep | 21.07 | 1,1599 | .000 | .013 |

*Note*. PA = political affiliation, PC = past political climate (i.e., 5 years prior to polling), Dem = Democrat, Rep = Republican.
