# Supplementary material for: Political Differences in Past, Present, and Future Life Satisfaction: Republicans Are More Sensitive than Democrats to Political Climate
Source: PLoS One. 2014 Jun 5;9(6):e98854. doi: 10.1371/journal.pone.0098854 (PMC4047066; doi:10.1371/journal.pone.0098854)
Supplement: Table S5 — Z scores for meta-analysis of combined significance tests. (DOCX) [file pone.0098854.s005.docx]

**Table S5. Z scores for meta-analysis of combined significance tests.**

| Model term | Covariate | DV | *F* | *p* | *Z* |
| --- | --- | --- | --- | --- | --- |
| PC | Yes | Present | 21.36 | .000002 | 4.611361 |
| PC | Yes | Future | 30.43 | <.000001 | 4.753408 |
| PC | Yes | Past (PC present) | 2.65 | .051805 | 1.627598 |
| PC | Yes | Past (PC past) | 5.64 | .008795 | 2.374137 |
| PC | No | Present | 25.73 | <.000001 | 4.753408 |
| PC | No | Future | 64.36 | <.000001 | 4.753408 |
| PC | No | Past (PC present) | 1.38 | .120074 | 1.174615 |
| PC | No | Past (PC past) | 4.92 | .013293 | 2.217543 |
| PA×PC | Yes | Present | 2.96 | .042703 | 1.720145 |
| PA×PC | Yes | Future | 6.61 | .005085 | 2.569995 |
| PA×PC | Yes | Past (PC present) | 7.55 | .003011 | 2.746578 |
| PA×PC | Yes | Past (PC past) | 3.76 | .026273 | 1.938633 |
| PA×PC | No | Present | 1.47 | .112697 | 1.212309 |
| PA×PC | No | Future | 10.17 | .000718 | 3.187313 |
| PA×PC | No | Past (PC present) | 9.97 | .000800 | 3.155904 |
| PA×PC | No | Past (PC past) | 15.51 | .000042 | 3.932690 |

*Note*. DV = dependent variable, PA = political affiliation, and PC = present political climate, except where DV indicates PC past. Probabilities are one-tailed as recommended by Rosenthal [43]. Where probabilities are less than .000001, that value was used to compute Z.
